# Customized Design 3D Printed PLGA/Calcium Sulfate Scaffold Enhances Mechanical and Biological Properties for Bone Regeneration

**DOI:** 10.3389/fbioe.2022.874931

**Published:** 2022-06-23

**Authors:** Tao Liu, Zhan Li, Li Zhao, Zehua Chen, Zefeng Lin, Binglin Li, Zhibin Feng, Panshi Jin, Jinwei Zhang, Zugui Wu, Huai Wu, Xuemeng Xu, Xiangling Ye, Ying Zhang

**Affiliations:** ^1^ General Hospital of Southern Theatre Command of PLA, The First School of Clinical Medicine, Southern Medical University, Guangzhou, China; ^2^ General Hospital of Southern Theatre Command of PLA, Guangzhou University of Chinese Medicine, Guangzhou, China; ^3^ Department of Trauma Orthopedics, Hospital of Orthopedics, General Hospital of Southern Theatre Command of PLA, Guangzhou, China; ^4^ The Fifth Clinical College of Guangzhou University of Chinese Medicine, Guangzhou, China; ^5^ Guangdong Key Lab of Orthopedic Technology and Implant Materials, General Hospital of Southern Theatre Command of PLA, Guangzhou, China; ^6^ Department of Orthopedics, Guangdong Second Traditional Chinese Medicine Hospital, Guangzhou, China

**Keywords:** bone defect, 3D printing scaffold, polylactic glycolic acid copolymer, calcium sulfate, mechanical properties, biological properties

## Abstract

Polylactic glycolic acid copolymer (PLGA) has been widely used in tissue engineering due to its good biocompatibility and degradation properties. However, the mismatched mechanical and unsatisfactory biological properties of PLGA limit further application in bone tissue engineering. Calcium sulfate (CaSO_4_) is one of the most promising bone repair materials due to its non-immunogenicity, well biocompatibility, and excellent bone conductivity. In this study, aiming at the shortcomings of activity-lack and low mechanical of PLGA in bone tissue engineering, customized-designed 3D porous PLGA/CaSO_4_ scaffolds were prepared by 3D printing. We first studied the physical properties of PLGA/CaSO_4_ scaffolds and the results showed that CaSO_4_ improved the mechanical properties of PLGA scaffolds. *In vitro* experiments showed that PLGA/CaSO_4_ scaffold exhibited good biocompatibility. Moreover, the addition of CaSO_4_ could significantly improve the migration and osteogenic differentiation of MC3T3-E1 cells in the PLGA/CaSO_4_ scaffolds, and the PLGA/CaSO_4_ scaffolds made with 20 wt.% CaSO_4_ exhibited the best osteogenesis properties. Therefore, calcium sulfate was added to PLGA could lead to customized 3D printed scaffolds for enhanced mechanical properties and biological properties. The customized 3D-printed PLGA/CaSO_4_ scaffold shows great potential for precisely repairing irregular load-bearing bone defects.

## 1 Introduction

Although bone is a tissue with superior self-healing potential, massive irregular bone defects created by trauma, tumor resection, or infection remain a challenge in the clinic (Kuss et al., 2017; Ye et al., 2018; Nulty et al., 2021; Yang et al., 2021; Ye et al., 2021). Autologous bone is considered an ideal material for the treatment of large bone defects due to its retention of osteoblasts and bioactive molecules, including growth factors with osteogenic induction properties (Cheng et al., 2018; Pförringer et al., 2018). However, there are some problems with autologous bone, such as limited donor bone and the risk of bleeding and infection during collection (Ishikawa et al., 2017; Lee et al., 2020). Allografts can also be used to treat bone defects to compensate for the limitations of autografts, but carry the risk of immune rejection (Lai et al., 2019). Thus, we needed to construct new materials as substitutes for autografts and allografts.

The success of load-bearing materials is largely dependent on physical and chemical properties that are known to drive cellular response and it is of great importance to construct a scaffold with an ability to promote cells proliferation, adhesion, migration, and differentiation for bone regeneration (Kim et al., 2017; Li H. et al., 2020). With the rapid development of bone tissue engineering, the interconnected porous scaffolds prepared by 3D printing technology to simulate the extracellular matrix of living bone are showing obvious advantages (Cui et al., 2018). More importantly, the 3D printing technology could fabricate custom-fit scaffolds based on a computed tomography scan of the defect site to repair irregular bone defects with complex geometry (Lai et al., 2019; Wu et al., 2021; Zhu et al., 2022). Besides, the customized design of 3D scaffolds can not only achieve the perfect match between the material and the bone defect but also regulate the structure of the material and the arrangement of cells in the microstructure, which is more conducive to promoting the growth and differentiation of cells and supporting the bone tissue regeneration process (Han et al., 2022).

In recent years, a variety of materials including polymers (Oryan and Sahvieh, 2017; Cui et al., 2019; Ranganathan et al., 2019; Lavanya et al., 2020; Hu et al., 2021; Yu et al., 2021), nanomaterials (Xia et al., 2018; Lu et al., 2019; Singh et al., 2019) metal materials (Lai et al., 2019; Tan et al., 2021), ceramic (Gao et al., 2017; Ma H. et al., 2018; Adithya et al., 2020), and other biological materials (Zimmermann and Ritchie, 2015; Daly et al., 2017; Bose and Sarkar, 2020; Cao et al., 2022; Pang et al., 2022) have been widely used in 3D printing technology to fabricate scaffolds for bone defect repair.

Among a variety of materials for bone tissue engineering, polylactic glycolic acid copolymer (PLGA) has been approved by US Food and Drug Administration (FDA) for human use due to its good biocompatibility and biodegradability (Jia et al., 2016). However, although PLGA is widely used in various tissue engineering applications, it still has the problems of mismatched mechanical and unsatisfactory biological properties owing to the low stiffness between PLGA-based implants and natural bones and the hydrophobic surface of PLGA-based scaffolds (Zou et al., 2020; Jin et al., 2021; Oizumi et al., 2021; Wei et al., 2021; Zhao et al., 2021). Many researchers address these problems by introducing inorganic material in the modification of PLGA-based scaffolds. To overcome the disadvantages of poor mechanical properties and osteogenic properties of PLGA, Zhu TT designed PLGA/nHA scaffolds to repair large bone defects and achieved good results (Zhu et al., 2022). Lai Y X constructed bone repair scaffolds by adding TCP to improve the mechanical properties of PLGA (Lai et al., 2019). In addition, PCL and bioglass are also used to improve the performance of PLGA (Cheng et al., 2018; Qian et al., 2019).

Calcium sulfate (CaSO_4_) is a commercial bone graft replacement material with a long history of application in a variety of medical applications, such as bone defect filling and tissue regeneration guidance. Calcium sulfate as a bone graft material has the advantages of the minimal inflammatory response, complete degradation, osteoconductive, and Ca^2+^ released during dissolution may promote osteogenic differentiation (Arun Kumar et al., 2016). As a synthetic bone graft material, CaSO_4_ could induce a biological reaction similar to that generated during bone remodeling, creating a calcium-rich environment in the implanted area (Zhou et al., 2014; Aquino-Martínez et al., 2017). Moreover, as an inorganic material, CaSO_4_ could enhance the mechanical strength and hydrophilicity of the polymeric scaffolds (Ye et al., 2018).

**SCHEME 1:**
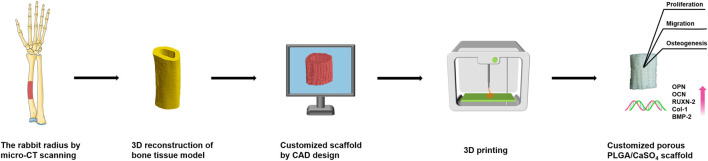
Schematic illustration of the fabrication process for PLGA/CaSO_4_ scaffolds. The rabbit radius was first scanned by micro-CT and a bone tissue model was constructed by 3D reconstruction. Then the scaffold was designed using CAD software, and the CAD data was transferred to a 3D printer to fabricate PLGA/CaSO_4_ scaffolds. The PLGA/CaSO_4_ scaffolds could promote cell proliferation, migration, and osteogenesis.

Herein we aim to develop 3D-printed customized scaffolds with proper mechanical and bioactivity properties for repairing irregular bone defects. In this study, we incorporated CaSO_4_ powder into PLGA and then fabricated 3D porous PLGA/CaSO_4_ scaffolds using fused deposition modeling (FDM) system (Scheme 1). The prepared PLGA, PLGA/10%CaSO_4_, PLGA/20%CaSO_4_, and PLGA/30%CaSO_4_ scaffolds all had a Customized 3D porous structure. We found that the addition of CaSO_4_ improved the mechanical properties of PLGA scaffolds, and with the increase of the CaSO_4_ ratio, the scaffolds could stand more pressure. Moreover, *in vitro* experiments showed that all scaffolds had good biocompatibility, and the PLGA/CaSO_4_ scaffolds improved the migration of MC3T3-E1 cells compared with PLGA scaffolds. Furthermore, PLGA/CaSO_4_ scaffolds significantly improved the osteogenic differentiation of MC3T3-E1 cells, and PLGA/20%CaSO_4_ scaffolds exhibited the best osteogenic properties. The customized-designed 3D porous PLGA/CaSO_4_ with satisfactory mechanical and proper biological are expected to solve the problems of PLGA scaffolds and be further used for irregular bone defects.

## 2 Materials and Methods

### 2.1 Materials

Polylactic glycolic acid copolymer (PLGA, MW = 15,000 g/mol) was purchased from Dai Gang Biology Co., Ltd. (Jinan, China). Anhydrous calcium sulfate (CaSO_4_, MW = 136.14 g/mol) was obtained from Macklin Biochemical Co., Ltd. (Shanghai, China).

### 2.2 Customized Design of Bone Defect Models

We scanned the rabbit radius bone by microcomputed tomography (micro-CT). Then the CT scan data of bone was imported into computer-aided design (CAD) software to establish a customized bone defect model, and generate the model file. The CAD data was utilized to design the scaffolds with the desired shape and 3D porous structure and then transferred to a 3D printer.

### 2.3 Fabrication of Customized 3D Polylactic Glycolic Acid Copolymer/CaSO_4_ Scaffold

The PLGA/CaSO_4_ scaffolds in different proportions were fabricated by a biological 3D printer (Livprint^®^ N series, Medprin, Guangzhou, China) layer by layer (Supplementary Figure S1). Firstly, PLGA and CaSO_4_ (CaSO_4_ accounts for 0, 10, 20, and 30 wt% of the quality of PLGA) powder were added into the beaker and then stirred evenly at 200°C (Du et al., 2018; Nulty et al., 2021). The mixture was then injected into the 3D printer and the scaffold was printed by following the constructed bone model. The nozzle temperature was 180°C, and the temperature of supporting substrates during FDM printing was 120°C.

### 2.4 Characterization of the Scaffolds

#### 2.4.1 Scanning Electron Microscopy Analysis

The surface morphology and pore size of the calcium sulfate powder and the scaffolds were observed using an Scanning Electron Microscopy (SEM) (Sigma 300, ZEISS, Germany). After being frozen in a refrigerator, freeze-dried in a lyophilizer, and coated with gold, the scaffolds were analyzed by SEM.

#### 2.4.2 Contact Angle

The hydrophilicity of each scaffold was measured using a contact angle measurement system (ASUMI GIKEN Limited, Tokyo, Japan). A droplet of deionized water was deposited on the scaffold. Then, the image of the static liquid deposition was obtained within a few seconds and the contact angles were measured. Three samples were assessed for each group to ensure reproducibility and the average value.

#### 2.4.3 Fourier Transform Infrared Spectroscopy Analysis

The Fourier transform infrared spectroscopy [(FTIR), Nicolet iS10, Thermo Fisher Scientific, United States] was used to evaluate the changes in the chemical structures of the scaffolds.

#### 2.4.4 X-ray Diffraction Analysis

X-ray diffraction (XRD) patterns were obtained using an Ultima IV X-ray diffractometer (Rigaku, Japan) in the range of 10°–80°.

#### 2.4.5 Mechanical Properties

The mechanical properties of the scaffold were evaluated by a universal machine (RGT-3, Shenzhen Reger Instrument Co., Ltd., China) with a constant speed of 1 mm/min. Three repeated measurements were made for each scaffold (Ye et al., 2018; Ma et al., 2020).

#### 2.4.6 Swelling Behavior

The swelling ratio of different scaffolds was weighed and placed in centrifuge tubes with 5 ml of simulated body fluid (SBF), and then placed on a shaker (37°C, 150 rpm/min). After 24 h, the scaffolds were taken out, removed the surface water by filter paper, and weighed. The swelling ratio was determined by using Eq. 1:

$$\text{Swell}\;\text{ratio}\left(\%\right)=\left({\text{W}}_{\text{w}}-{\text{W}}_{\text{d}}\right)/W_{d}\times 100\%$$ (1)

where *W*_*w*_ and *W*_*d*_ are the wet and dry weights, respectively.

#### 2.4.7 Biodegradation

The initial weight of each scaffold was recorded and placed in a centrifuge tube containing 5 ml of SBF. The tubes were placed in a shaker stirring at a speed of 150 rpm at 37°C. Scaffolds were removed from the tubes every 3 days and weighted, and then replaced with the SBF solution (Li et al., 2021). The percentage of degradation was calculated using Eq. 2:

$$\text{Degradation}\left(\%\right)={\text{W}}_{\text{t}}/{\text{W}}_{\text{i}}\times 100\%$$ (2)

Where *W*_*i*_ is the initial weight of the samples and *W*_*t*_ is the weight at each time interval.

### 2.5 Biocompatibility *In Vitro*

#### 2.5.1 Cell Culture

Human umbilical vein endothelial cells (HUVECs) were purchased from Cyagen Biotechnology (United States), and cultured with Dulbecco’s Modified Eagle’s Medium (DMEM high glucose, Gibco, United States) containing 10% fetal bovine serum (FBS, Biological Industries, United States) and 1% penicillin-streptomycin (Gibco, United States) at 37°C in a humidified and 5% CO_2_ incubator.

#### 2.5.2 Cell Proliferation

Cell Counting Kit-8 (CCK-8) assay was used to evaluate the cell viability for proliferation in the scaffold. HUVEC cells were seeded in a 24-well plate at a density of 1 × 10^4^ cells per well and incubated with different scaffolds for 1, 2, and 3 days, respectively (Kim DS et al., 2021). The scaffolds were removed from the plate and the media solution was replaced with 300 μl CCK-8 solution (Biosharp, China) in each well and incubated for 2 h. 100 μL of the supernatant was removed to a 96-well plate and the OD value was measured with a microplate (Multiskan GO, Thermo Fisher Scientific, United States).

#### 2.5.3 Live/Dead Cell Staining

Live/dead staining was used to evaluate the cytocompatibility of the scaffolds. The HUVECs were co-cultured with different scaffolds for 1, 2, and 3 days. After being washed with PBS, Calcein-AM/palladium staining solution (Bestbio, China) was added to each well for 30 min at room temperature. A fluorescence microscope (DMI4000, Leica, Germany) was employed to record the fluorescent images of HUVEC cells.

#### 2.5.4 Hemolysis Tests

Healthy human blood containing EDTA was collected and diluted with PBS in a ratio of 4:5. Then the different scaffolds were immersed in 1.8 ml of PBS in each group and incubated at 37°C for 30 min, and 2 ml of ddH_2_O and PBS were set as positive and negative controls, respectively. Next, 0.2 ml of the diluted whole blood was added to each scaffold sample, and the scaffolds were incubated at 37°C for 1 h. Then, the samples were centrifuged at room temperature (3,000 rpm, 5 min). The supernatant was removed from the samples and the absorbance at 545 nm was measured with a microplate reader (Ye et al., 2018). The hemolysis rate (HR) was calculated using Eq. 3:

$$\text{HR}\left(\%\right)=\left(\text{O}{\text{D}}_{\text{s}}-\text{O}{\text{D}}_{\text{n}}\right)/\left(\text{O}{\text{D}}_{\text{p}}-\text{O}{\text{D}}_{\text{n}}\right)\times 100\%$$ (3)

where *OD*_*s*_, *OD*_*p*_, and *OD*_*n*_ are the *OD* values of the scaffold, positive control, and negative control groups, respectively.

### 2.6 Cell Migration and Adhesion

#### 2.6.1 Cell Culture

Mouse embryo osteoblast precursor cells (MC3T3-E1) were purchased from Cyagen Biotechnology (United States), and cultured with Minimum essential medium alpha (MEM-α, Gibco, United States) containing 1% penicillin-streptomycin and 10% fetal bovine serum at 37°C in a humidified and 5% CO_2_ incubator.

#### 2.6.2 Wound Healing Assay

For the cell migration assay, MC3T3-E1 cells were seeded in 12-well plates at a density of 5 × 10^4^ cells per well. After the cells were cultured to confluence, a straight scratch was made with a 200 μl pipette tip, and then the scaffolds were directly co-culture with MC3T3-E1 cells for 12 h and stained with Calcein-AM/PI kit for 30 min at room temperature. After removing the free dyes, the distance of the scratch was visualized with a fluorescence microscope and the wound healing rate was calculated using Eq. 4:

$$\text{Cell}\;\text{migration}\;\text{rate}\left(\%\right)=\left({\text{A}}_{0\text{h}}-{\text{A}}_{24\text{h}}\right)/{\text{A}}_{0\text{h}}\times 100$$ (4)

where *A*_*0h*_ and *A*_*24h*_ are the initial distance and the gap after 24 h of coculture, respectively.

#### 2.6.3 Transwell Migration Assay

The migration of MC3T3-E1 cells was also tested using a transwell assay. Briefly, 80 μl of Matrigel (Corning, United States) was added to the upper chambers of a 24-well transwell plate (Corning; pore size = 8 µm) and gelatinized for 2 h at 37°C. The scaffolds were completely immersed in MEM-α culture medium, which contained 10% FBS, and 1% penicillin-streptomycin at a concentration of 10 mg/ml. The samples were maintained in a shaker at 37°C with a speed of 120 rpm to obtain the extract solutions. 600 μl extracted liquid from each scaffold containing 20% FBS was added to the lower chamber. Then, MC3T3-E1 were seeded in the upper chambers at a density of 1 × 10^5^ cells per well. After incubation at 37°C for 24 h, the Matrigel was erased with a swab, and MC3T3-E1 migrated to the opposite side of the membrane were fixed with 4% paraformaldehyde for 30 min and stained with 0.5% crystal violet (Macklin, China) for 1 h. Three random fields from each plate were recorded using an optical microscope. The stained MC3T3-E1 were lysed in 95% ethanol for 1 h to measure the OD value at 590 nm using a microplate reader.

#### 2.6.4 Cytoskeleton Analysis

Cytoskeleton staining was used to evaluate cell morphology on the scaffold surface. Briefly, MC3T3-E1 cells were incubated with different scaffolds at a density of 1 × 10^4^ per well in a 24-well plate. After incubation for 3 days, cells were fixed with 4% paraformaldehyde for 2 h and then permeabilized with 0.1% Triton X-100 (Sigma-Aldrich, United States) for 5 min at room temperature. After washed with PBS, the cells were stained with Actin Cytoskeleton/Focal Adhesion Staining Kit (FAK 100, Sigma-Aldrich, United States) for 1 h and DAPI (Solarbio, China) for 5 min at room temperature. The reaction was stopped by removing the DAPI solution and washing it with PBS, the cells were visualized with confocal laser scanning microscopy (CLSM) (TCS SP-2, Leica, Germany).

### 2.7 Osteogenic Activity *In Vitro*

#### 2.7.1 Alkaline Phosphatase Staining

The Alkaline Phosphatase (ALP) activity assay was performed to analyze the effect of scaffolds on the early osteogenic differentiation of cells. For ALP staining, MC3T3-E1 cells were seeded in a 6-well plate at a density of 1 × 10^4^ cells per well and incubated with different scaffolds for 7 and 14 days, respectively. The ALP activity assay was then performed with the BCIP/NBT alkaline phosphatase color development kit (Beyotime, China) according to the manufacturer’s instructions. After removing the ALP stain working solution and washing with PBS. Stained MC3T3-E1 cells were visualized with an inverted research microscope (DMI4000, Leica, Germany).

#### 2.7.2 Alkaline Phosphatase Activity

The ALP activity was also employed to evaluate the effect of scaffolds on the osteogenic differentiation of cells. MC3T3-E1cells were seeded in a 6-well plate at a density of 1 × 10^4^ cells per well and incubated with different scaffolds for 7 and 14 days, respectively. After the incubation, cells were washed with PBS and lysed using 0.2% Triton X-100 for 12 h at 4°C. ALP activity was determined using an ALP detection kit (P0321, Beyotime Biotechnology, China). The total protein content in the samples was determined by the BCA protein assay kit (Thermo Fisher Scientific, United States) with the same protocol above. The relative ALP activity was finally normalized to the corresponding total protein content.

#### 2.7.3 Alizarin Red Staining

For the Alizarin red assay, MC3T3-E1 cells were fixed with 4% paraformaldehyde for 30 min at room temperature after culturing with scaffolds for 7 and 14 days. Then the cells were stained with 400 μl Alizarin red S solution (ARS, Sigma, United States) for 3 h. The plates were then observed with a microscope.

#### 2.7.4 Gene Expression

MC3T3-E1 cells were seeded in a 6-well plate at a density of 1 × 10^4^ cells per well and incubated with different scaffolds for 7 and 14 days, respectively. The osteogenesis-related genes include osteoprotein (OPN), osteocalcin (OCN), runt-related transcription factor 2 (RUNX2), type I collagen (COL-1), and bone morphogenetic protein-2 (BMP-2) were analyzed by real-time quantitative polymerase chain reaction (RT-qPCR). Total RNA was obtained from the MC3T3-E1 cells with a total RNA extraction kit (Accurate Biology, China) and reversed transcribed into complementary DNA with the PrimeScript TM reagent kit (Takara, Japan). The gene expression levels were quantified using an ABI Prism 7000 machine (Thermo Fisher Scientific, United States) with TB Green Premix Ex Taq II (Takara, Japan). Primers were presented in Supplementary Table S1.

### 2.8 Statistical Analysis

Analysis was performed using SPSS 19.0 software (IBM, United States). A two-tailed Student’s t-test was used in the comparison between the two groups. One-way analysis of variance (ANOVA) followed by Tukey’s multiple comparisons was carried out in the comparison among more than two groups (*p < 0.05, **p < 0.01, and ***p < 0.005). All the data are expressed with mean ± standard deviation.

## 3 Results

### 3.1 Preparation and Characterization of the Scaffolds

**FIGURE 1:**
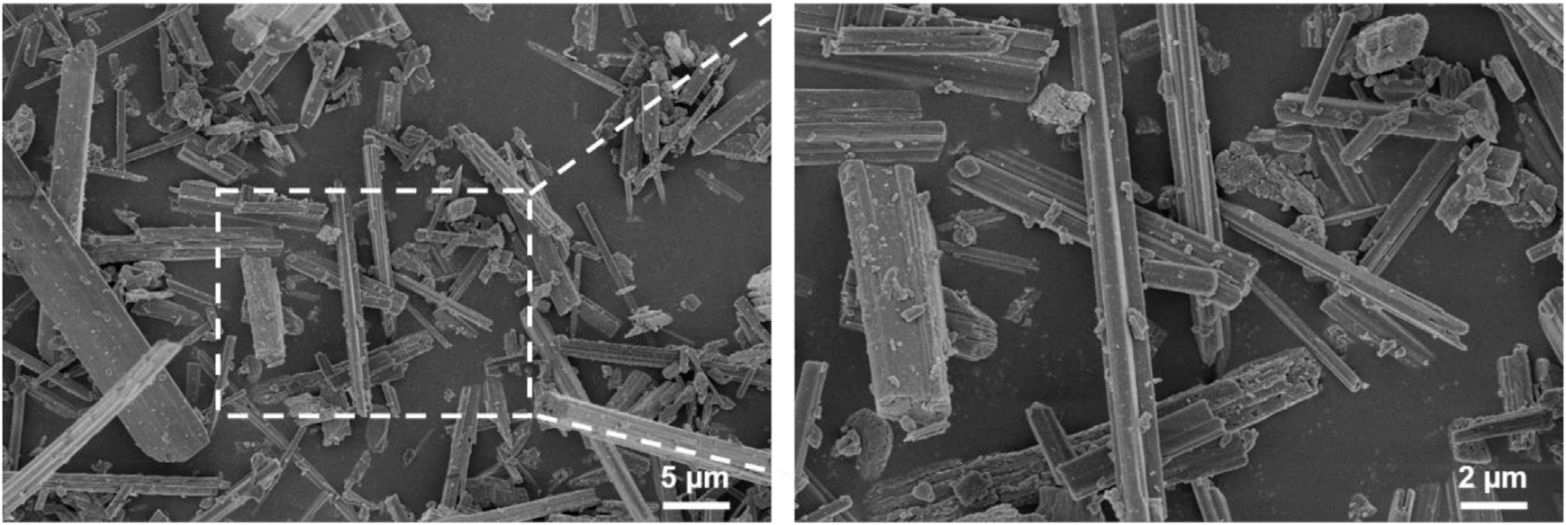
SEM morphology of CaSO_4_ particles.

The shape, size, and morphology of the CaSO_4_ particles are closely related to the formation of 3D printed scaffolds. The morphology of CaSO_4_ particles was evaluated using SEM. As shown in Figure 1, the particle size of CaSO_4_ was about 2.0–20.0 µm, and the particles were evenly dispersed without agglomeration. CaSO_4_ particles are a new type of fiber with high mechanical strength compared with polymers due to the near-perfect crystal structures (Fm et al., 2021), so they could be used as a potential reinforcement material for PLGA.

**FIGURE 2:**
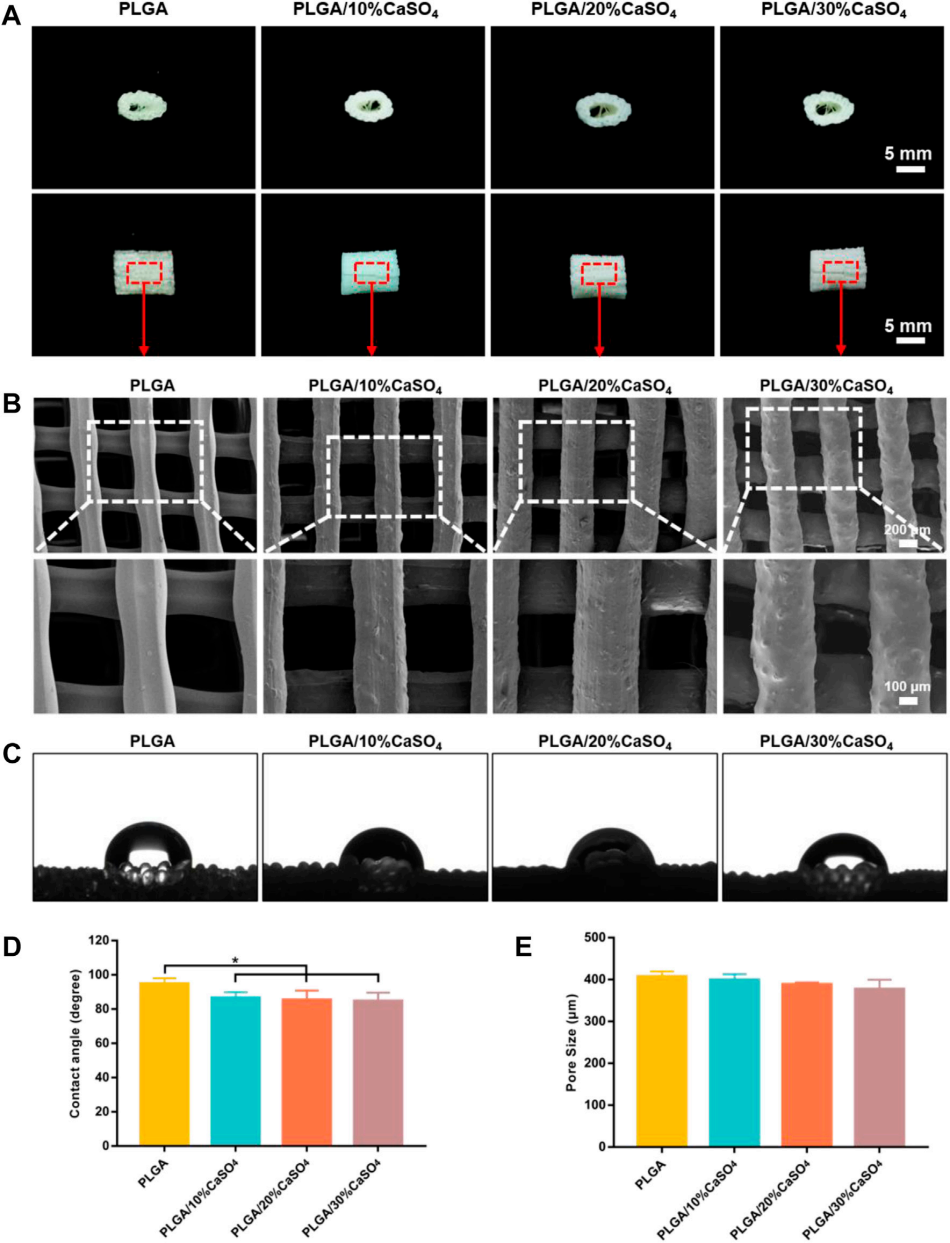
Characterization of the scaffolds. **(A)** Representative pictures of 3D printed PLGA, PLGA/10%CaSO_4_, PLGA/20%CaSO_4_, and PLGA/30%CaSO_4_ scaffolds. Scale bar = 5 mm. **(B)** SEM images of the side of the different scaffolds. Scale bar = 200 and 100 μm, respectively. **(C)** Water contact angle images of different scaffolds. **(D)** Contact angle (degree) of PLGA, PLGA/10%CaSO_4_, PLGA/20%CaSO_4_, and PLGA/30%CaSO_4_ scaffolds, respectively. **(E)** The pore size of each scaffold. Data are presented as mean ± SD (*n* = 3); *p < 0.05, **p < 0.01, ***p < 0.001.

We successfully constructed customized-designed PLGA, PLGA/10%CaSO_4_, PLGA/20%CaSO_4_, and PLGA/30%CaSO_4_ scaffolds by a 3D printer in the FDM system, respectively (Figure 2A). SEM was employed to observe the microstructure and surface morphology of the scaffolds. As shown in Figure 2B, all the scaffolds had a three-dimensional network structure and there was no statistical difference between the pore size of different scaffolds (Figure 2E). The scaffolds had a regular structure with interconnected pores of about 400 μm. The interconnected macropores facilitate the diffusion of oxygen and nutrients, providing sufficient space for the proliferation, adhesion, migration, and differentiation of cells (Zhao et al., 2018; Yan et al., 2019; Gu et al., 2021; Kim H. D. et al., 2021). High-resolution SEM showed that the surface of the pure PLGA scaffold was smooth. However, with the incorporation of calcium sulfate, the roughness of the surface was improved (Sivashanmugam et al., 2017), which facilitated cell adhesion and migration (Kim D.-S. et al., 2021).

The water contact angle test was performed to evaluate the surface hydrophilicity of the scaffolds. Figure 2C showed the contact angle images of each scaffold. Due to the intrinsic hydrophobicity of PLGA (Zhu et al., 2022), the water contact angle of the PLGA scaffold was (94.88 ± 3.20°), while the contact angle of PLGA/10%CaSO_4_, PLGA/20%CaSO_4_, and PLGA/30%CaSO_4_ were (86.57 ± 3.30°), (85.33 ± 5.47°), and (84.75 ± 4.91°), respectively (Figure 2D). The incorporation of CaSO_4_ decreased the contact angle of the scaffolds. Thus, CaSO_4_ improved the hydrophilicity of the composite scaffold. Considering that the hydrophilicity of the materials played an important role in protein absorption and cell proliferation (Liu et al., 2018), the improvement in the hydrophilicity of the PLGA/CaSO_4_ scaffold may determine the subsequent cellular behavior.

**FIGURE 3:**
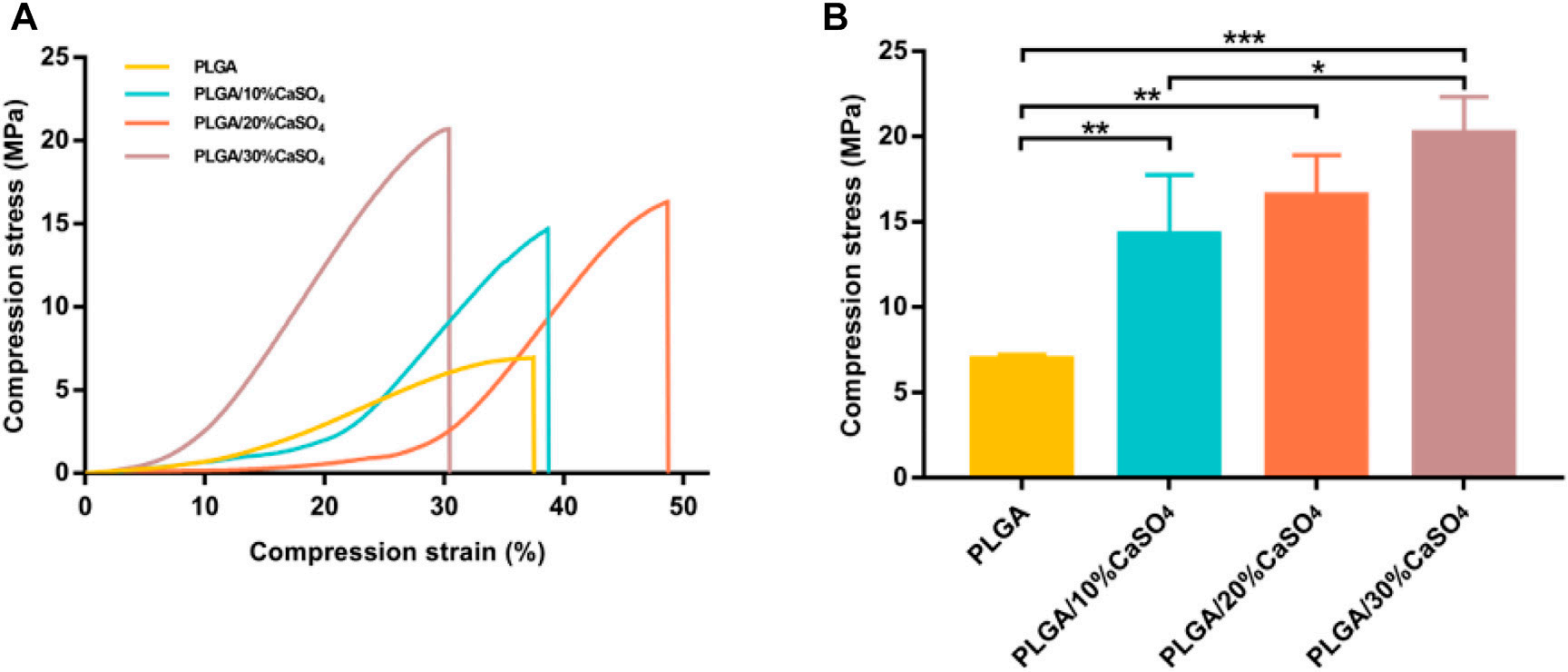
Mechanical performance of the 3D printed scaffolds. **(A)** The stress-strain curves. **(B)** The compress stress. Data are presented as mean ± SD (*n* = 3); *p < 0.05, **p < 0.01, ***p < 0.001.

Excellent mechanical properties are essential for scaffolds. Hence, we performed compress tests on different groups of scaffolds. As shown in Figure 3A, PLGA scaffolds had minimum compress stress. Figure 3B showed the compressive strength of the scaffolds. The compressive strength of the PLGA scaffold was 6.95 MPa. After adding different CaSO_4_ content, the compression stress of the scaffolds had improved, which were 14.27, 16.54, and 20.21 MPa, respectively. The scaffolds made with 30% CaSO_4_ exhibited maximum compressive stress. When CaSO_4_ is combined with H_2_O, a hydration reaction can occur to form needle-like calcium sulfate dihydrate whiskers. These whiskers bridge and stack with each other to solidify into deposits of a certain shape and strength, which has better mechanical properties (Chiang et al., 2021). Therefore, CaSO_4_ could enhance the mechanical properties when combined with polymer materials, enable the material to withstand greater deformation, and give it better mechanical properties (Zhao et al., 2008). The result indicated that the addition of CaSO_4_ could greatly enhance the mechanical properties of the PLGA scaffold to bear loads.

The changes in the chemical structure of the composite before and after the addition of CaSO_4_ could be observed in the FTIR spectra. As shown in Supplementary Figure S2. PLGA spectrum showed an intense peak of characteristic carbonyl (C=O) at 1,850 cm^−1^. In the same spectrum could also be observed a characteristic peak of the C-C(=O)-O at 1,150 cm^−1^ (Fernandes et al., 2017; Chen et al., 2021). After adding CaSO_4_, the PLGA/CaSO_4_ peaks became less intense and narrow, showing a decrease in the PLGA ratio. The XRD patterns (Supplementary Figure S3) of the PLGA/CaSO_4_ scaffolds showed characteristic crystalline peaks at 15, 25, 30, 31, and 48° corresponding to (200), (020), (002), (102), and (302) planes of CaSO_4_ (Sindhura et al., 2014; Zhu et al., 2022). These characteristic peaks of the CaSO_4_ in the scaffolds intensified with the increasing proportion of the CaSO_4_, thereby indicating the successful doping of the CaSO_4_ into the PLGA/CaSO_4_ scaffold. The swelling ratio represents the ability of the material to absorb water. As shown in Supplementary Figure S4, the swelling ratio of the different scaffolds was similar and had no statistical difference (*p* > 0.05), indicating that the addition of CaSO_4_ could not change the swelling of the scaffolds.

### 3.2 The *In Vitro* Degradation

**FIGURE 4:**
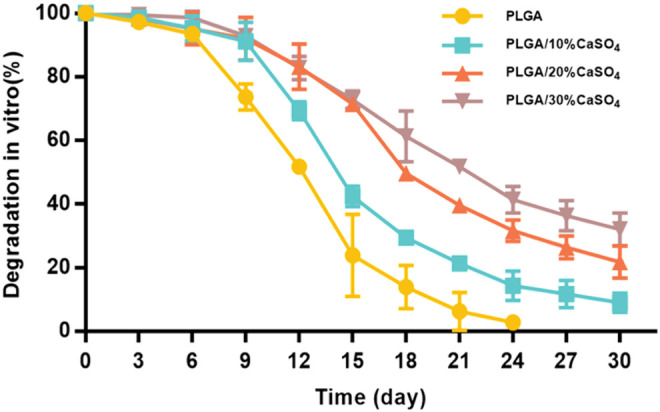
Degradation *in vitro*.

The biodegradability of materials is highly beneficial for clinical applications since it could be able to prevent damage caused by secondary surgical removal (Li et al., 2021). An ideal biomimetic scaffold composed of biodegradable materials should provide proper mechanical support while degrading to non-toxic products being excreted from the body ultimately. The weight loss of scaffolds in SBF was evaluated to study the *in vitro* degradation (Figure 4). We found that all scaffolds degrade slowly in the first 6 days, which meant that the scaffold could provide effective support and protection for bone in the initial stage of bone defect repair (Qian et al., 2014; Tan et al., 2021; Xue et al., 2021). Subsequently, the degradation rate of the scaffolds accelerated in all groups. PLGA degraded fastest and could be completely degraded within 24 days, which was inconsistent with the rate of bone repair. This indicated that PLGA could not be used as a scaffold alone to repair bone defects (Qian et al., 2014; Jia et al., 2016; Lai et al., 2019; Dos Santos et al., 2020; Kim H. D. et al., 2021). The degradation rate of PLGA/CaSO_4_ scaffolds was decreased than that of PLGA. PLGA was completely degraded in 4 weeks and the degradation products were polylactic acid and glycolic acid. CaSO_4_ was difficult to dissolve in water and it can combine with H_2_O to form calcium sulfate whiskers (Chiang et al., 2021). The whiskers were interconnected and stacked together to make their structure even tighter, which made CaSO_4_ have a relatively slow degradation rate and can be completely degraded within 6 weeks. What’s more, CaSO_4_ mixed with PLGA can form a dense solid, which can slow down the degradation rate (Amirthalingam et al., 2021). The addition of CaSO_4_ slowed down the degradation rate of PLGA (Arun Kumar et al., 2016). This indicated that CaSO_4_ improved the degradation performance of PLGA scaffolds. The bone-bonding ability and *in vivo* bone bioactivity of bone repair materials could be evaluated by examining the ability of apatite to form on its surface in SBF (Kokubo and Takadama, 2006). Chan et al. (2004) observed that calcium sulfate could form apatite on the surface both in SBF and *in vivo*. The findings indicated that calcium sulfate precipitation as carbonate-containing hydroxyapatite and its surface apatite formation in SBF could enhance the acellular and bone bioactivity. Hence, the addition of CaSO_4_ would promote the formation of apatite on the scaffolds and enhance the acellular bioactivity.

### 3.3. *In Vitro* Biocompatibility

**FIGURE 5:**
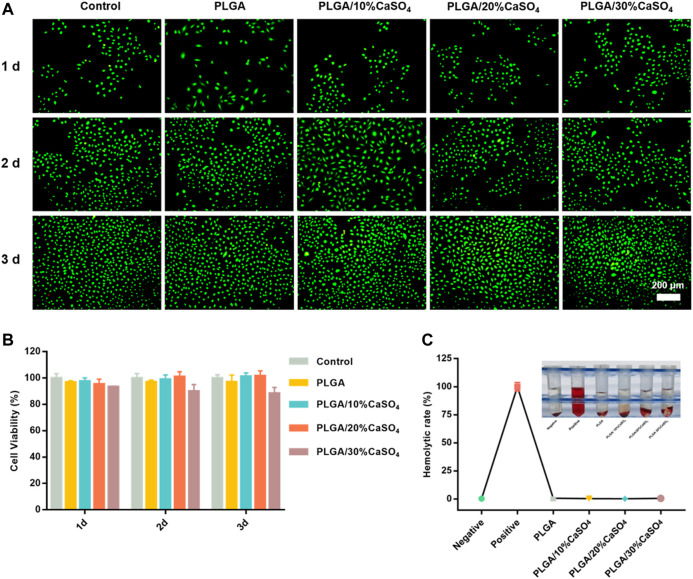
*In vitro* biocompatibility. **(A)** Representative fluorescence images for HUVEC cells cultured with different scaffolds. Live cells were stained by calcein-AM (green color). Scale bar = 200 μm. **(B)** CCK-8 assay for HUVECs cultured with the PLGA, PLGA/10%CaSO_4_, PLGA/20%CaSO_4_, and PLGA/30%CaSO_4_ scaffolds, respectively. **(C)** *In vitro* hemolysis of different scaffolds and Hemolytic rate (%). Data are presented as mean ± SD (*n* = 3); *p < 0.05, **p < 0.01, ***p < 0.001.

Biocompatibility is an important indicator for the clinical use of biomaterials in orthopedics. Therefore, biocompatibility assessment is an important part of biomedical materials and a primary requirement for the development of biomaterials. First, HUVECs proliferation in different scaffolds was evaluated by CCK-8 assay. As shown in Figure 5B, the scaffolds had good cell viability after incubating with HUVEC cells for 1, 2, and 3 days. However, PLGA/30%CaSO_4_ scaffolds had the lowest cell viability rate. This might be because Ca^2+^ was the second messenger in cells, and excessive Ca^2+^ affects cell signaling, thereby inhibiting cell proliferation and migration (Teparat-Burana et al., 2015). We further evaluated the biocompatibility by live/dead staining assay (Figure 5A). HUVEC cells indicated by green fluorescence were still alive after incubating with different scaffolds for 1, 2, and 3 days. The number of cells in control, PLGA, PLGA/10%CaSO_4_, PLGA/20%CaSO_4_, and PLGA/30%CaSO_4_ groups increased with the culturing time, suggesting that the scaffolds had non-toxicity to HUVEC cells. Hemolysis rate is another indicator to evaluate the biocompatibility of materials (Li et al., 2021). Hemolysis experiment results showed that the supernatant in the positive control group turned red because the relatively low osmotic pressure caused a large number of erythrocytes to rupture (Figure 5C) (Li et al., 2021). While the supernatant in the negative control group and the scaffold groups was still clear, demonstrating that almost no red blood cells were broken. In addition, the hemolysis ratio of the different scaffolds was less than 5%, which met the requirements for the hemolysis rate of medical materials (Tang et al., 2020). These observations suggested that PLGA, PLGA/10%CaSO_4_, PLGA/20%CaSO_4_, and PLGA/30%CaSO_4_ scaffolds had good biocompatibility.

### 3.4 *In Vitro* Cell Migration and Adhesion

**FIGURE 6:**
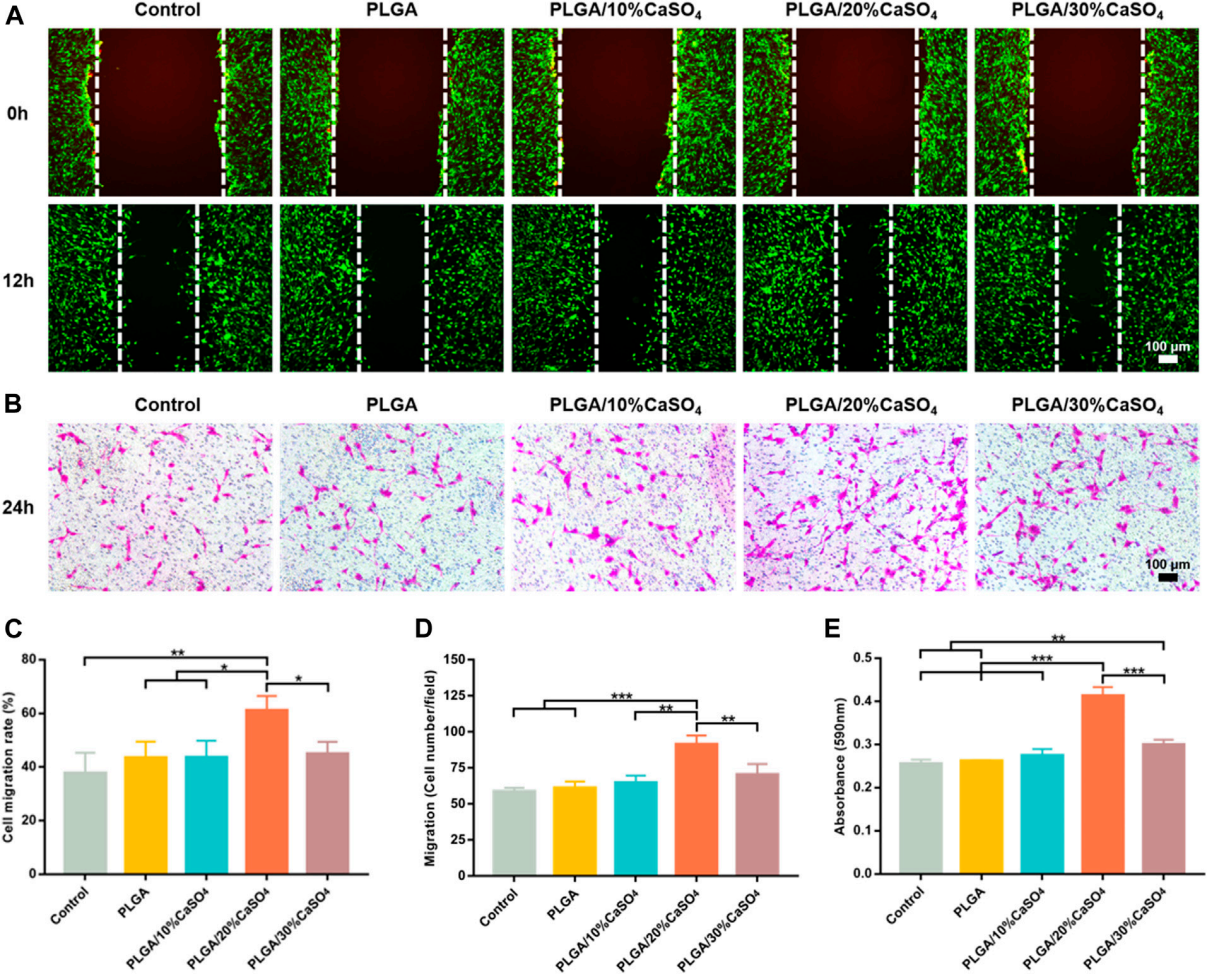
*In vitro* cell migration. **(A)** *In vitro* wound healing assay of MC3T3-E1 cell. Scale bar = 100 μm. **(B)** Transwell assay of MC3T3-E1 cells. Scale bar = 100 μm. **(C)** Cell migration rate (%) of cells. **(D)** Cell migration number of MC3T3-E1 cells per field. **(E)** Absorbance at 590 nm. Data are presented as mean ± SD (*n* = 3); *p < 0.05, **p < 0.01, ***p < 0.001.

The repair of bone defects depends on the proliferation and migration of cells, so the ideal bone repair material should be able to promote the migration of osteoblasts. Wound healing assay and transwell assay were used to simulate the effect of scaffolds on osteoblast migration. As shown in Figure 6A, compared with control, PLGA, PLGA/10%CaSO_4_, and PLGA/30%CaSO_4_ groups, PLGA/20%CaSO_4_ could significantly promote MC3T3-E1 cells migration. Figure 6C showed that the PLGA/20%CaSO_4_ group had the highest cell migration rate (61.16%). The transwell assay (Figure 6B) also showed that PLGA/20%CaSO_4_ group had the highest number of cell migrations per field (Figure 6D). What’s more, compared with other groups, PLGA/20%CaSO_4_ group had a higher OD value (Figure 6E). Calcium sulfate as a bone graft material had osteoconductive properties, completely degradable. CaSO_4_ promotes cell migration in a concentration-dependent manner. It recruits cells to migrate to sites with high concentrations of CaSO_4_. But CaSO_4_ to promote cell migration requires a suitable concentration range. When calcium levels exceed this range, cell migration is inhibited (Arun Kumar et al., 2016; Aquino-Martínez et al., 2017). What’s more, excessive calcium ions will affect cell proliferation, and cell migration will also be affected when the number of cell proliferation is reduced (Teparat-Burana et al., 2015), which was consistent with the results of *in vitro* biocompatibility. The PLGA/20%CaSO_4_ scaffold had the highest cell viability, while the cell viability in PLGA/30%CaSO_4_ decreased compared with 20%. Therefore, scaffolds in the 20% group had a strong ability to promote cell migration, which plays a critical role in bone reconstruction.

**FIGURE 7:**
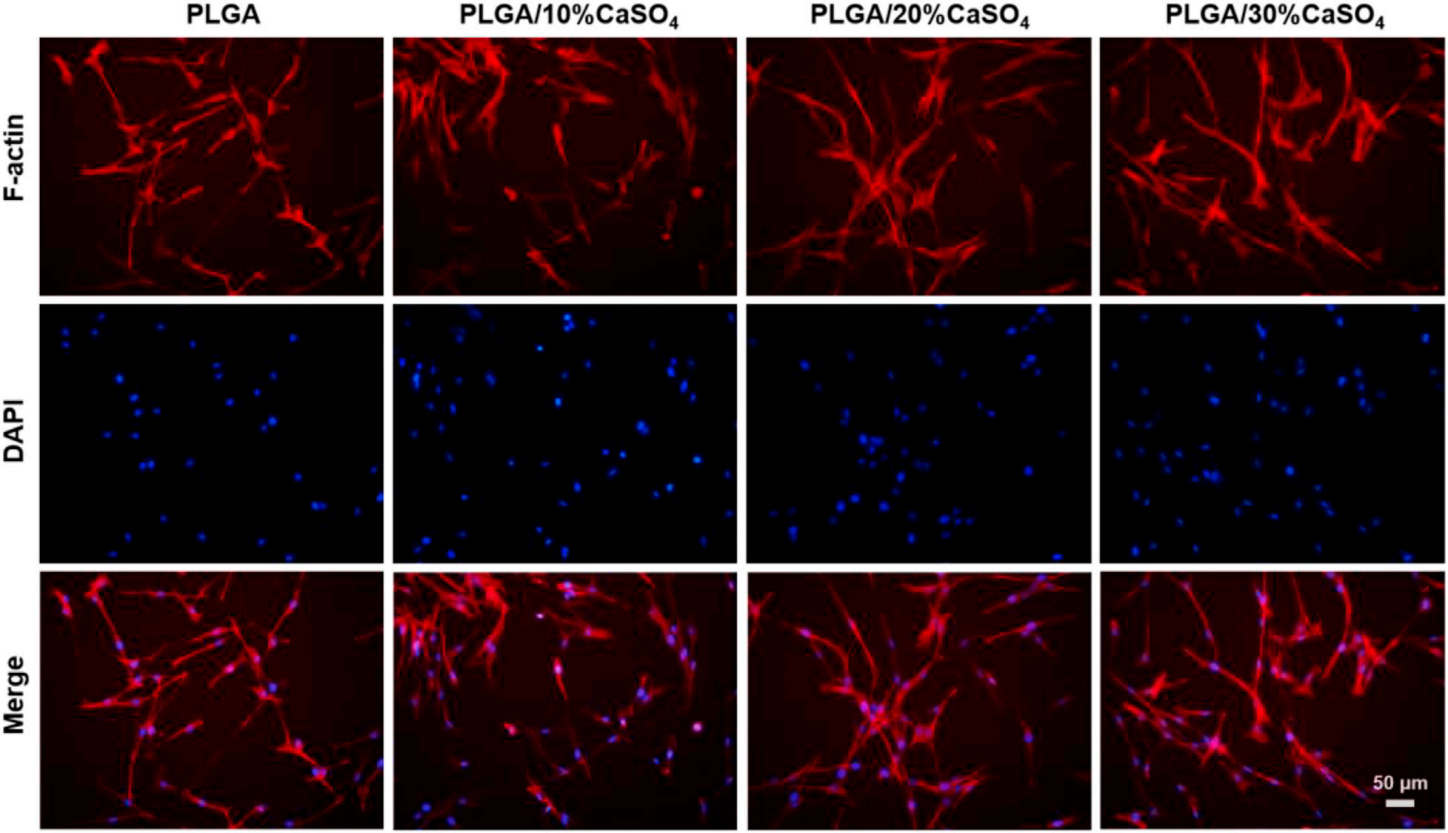
MC3T3-E1 cytoskeleton in the scaffolds for 24 h. Scale bar = 50 μm.

We also carried out the cytoskeleton staining to evaluate the cell extension and adhesion on the scaffolds (Figure 7), which showed spindle MC3T3-E1 cells presented well-stretch morphology and favorable proliferation status on PLGA/10%CaSO_4_, PLGA/20%CaSO_4_, and PLGA/30%CaSO_4_ scaffolds than on PLGA scaffold. Moreover, the number of adhered cells on the surface of the PLGA/10%CaSO_4_, PLGA/20%CaSO_4_, and PLGA/30%CaSO_4_ scaffolds were more than that of PLGA scaffolds, and the number and distribution density of actin microfilaments in the cytoskeleton were also more than those on the surface of PLGA scaffolds. Spreading and differentiation of cells were particularly affected by microscopic roughness and hydrophilicity. Hydrophilic biomaterial surfaces could promote cell growth and improve biocompatibility. Besides, CaSO_4_ could promote cell proliferation, extension, and adhesion (Phang et al., 2004). Thus, the PLGA/CaSO_4_ scaffold constructed by adding CaSO_4_ with PLGA had good properties to promote cell expansion and adhesion due to the improvement of surface hydrophilicity.

### 3.5 Evaluation of Osteogenic Differentiation *In Vitro*

**FIGURE 8:**
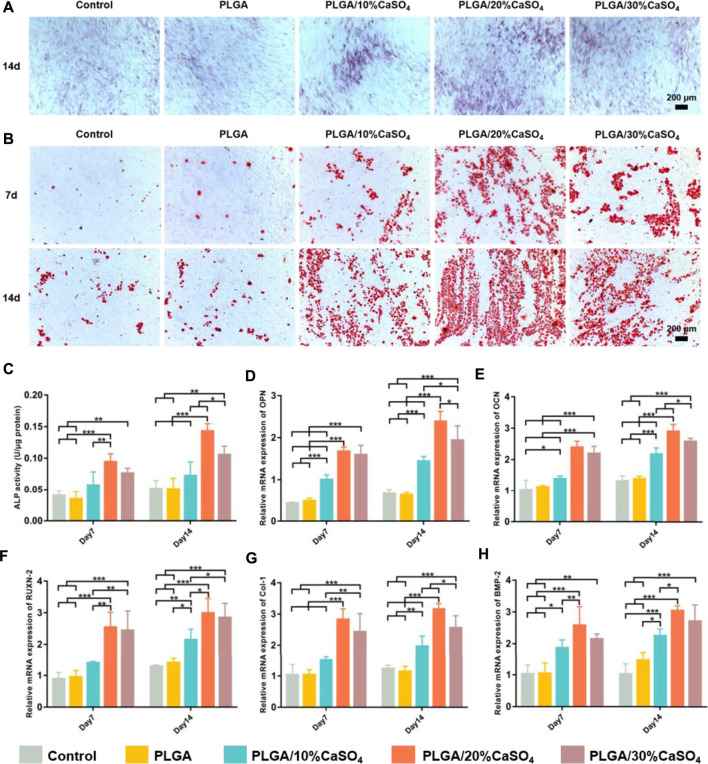
*In vitro* osteogenesis capability of the scaffolds. **(A)** ALP staining. Scale bar = 200 μm. **(B)** Staining area of Alizarin red. Scale bar = 200 μm. **(C)** The ALP activity of the MC3T3-E1 cells co-cultured with the scaffolds on days 7 and 14. ALP level was significantly high in the PLGA/20%CaSO_4_ scaffolds compared to the other scaffolds. **(D–H)** Relative mRNA expression of the osteogenic genes (OPN, OCN, RUNX-2, Collagen I, and BMP-2). Data are presented as mean ± SD (*n* = 3); *p < 0.05, **p < 0.01, ***p < 0.001.

Osteogenic differentiation property is the key to evaluating the success of a biomaterial (Huan and Chang, 2007; Ma L. et al., 2018; Wang et al., 2018; Freeman et al., 2019). We detected the osteogenic differentiation of the different scaffolds based on ALP staining, ALP activity assay, alizarin red staining, and osteogenesis-related genes expression. As an important early osteogenic enzyme during osteogenesis, the activity of ALP was represented as a typical marker of osteogenic differentiation (Yin et al., 2019; Li M. et al., 2020; Zhai et al., 2021). ALP staining result was shown in Figure 8A. It was found that the PLGA/20%CaSO_4_ exhibited deeper dyeing than other groups on day 14. This indicated that more ALP was produced in the PLGA/20%CaSO_4_ group. The ALP activity of the MC3T3-E1 cells in the PLGA/20%CaSO_4_ and PLGA/30%CaSO_4_ groups was significantly higher than in other groups on day 7 (Figure 8C). And the ALP activity in the PLGA/20%CaSO_4_ group was significantly higher than in other groups on day 14. Therefore, compared with other groups, PLGA/20%CaSO_4_ group can effectively promote the production of ALP, which was consistent with the above results of ALP staining. Figure 8B showed the results of Alizarin staining on days 7 and 14. Compared with the control group and PLGA group, PLGA/10%CaSO_4_, PLGA/20%CaSO_4_, and PLGA/30%CaSO_4_ group scaffolds could promote the production of calcium nodules. Cells cultured with PLGA/20%CaSO_4_ scaffold had the largest red staining area and the most calcium nodules compared with the other groups.

Moreover, we examined the expression levels of several critical osteogenic genes including OPN, OCN, RUNX-2, Collagen I, and BMP-2. As shown in Figures 8D–H, the expression levels of BMP-2, Col-1, RUXN-2, and OCN genes in PLGA/20%CaSO_4_ and PLGA/30%CaSO_4_ groups were higher than in other groups on day 7, and the expression level of OPN in the PLGA/20%CaSO_4_ group was the highest at day 7. And the expression levels of all these genes in the PLGA/20%CaSO_4_ group were higher than in other groups on day 14. These results suggested that CaSO_4_ could promote osteogenic differentiation *in vitro*, which could make up for the lack of osteogenic induction activity of PLGA (Du et al., 2018; Kim D.-S. et al., 2021). And PLGA/20%CaSO_4_ scaffold had the best osteogenic performance among all the scaffolds we constructed.

Calcium sulfate is the most commonly used bone repair material in clinical, which can cause changes in local calcium ions concentration after implantation, thereby regulating the process of bone tissue regeneration. As an intracellular second messenger and an important signaling molecule, Ca^2+^ controls many key processes in cells, including proliferation, migration, and differentiation (Teparat-Burana et al., 2015). Ca^2+^ is released during CaSO_4_ degradation Ca^2+^ can bind to calmodulin (CaM) to Ca^2+^-CaM form complexes and promote osteoblast differentiation and matrix mineralization through corresponding signaling pathways (Cao et al., 2022). The favorable osteogenic property of CaSO_4_ could improve the insufficient osteoinductive activity of polymer materials (Du et al., 2018; Kim H. D. et al., 2021). As mentioned above, we think that the incorporation of CaSO_4_ in the PLGA scaffolds provides Ca^2+^ ions that might enhance the osteogenic differentiation of cells. In addition, the prepared PLGA/CaSO_4_ scaffolds improved the hydrophilicity of the scaffold surface, which was more conducive to cell proliferation and adhesion on the scaffold, and further promoted osteogenic differentiation.

## 4 Conclusion

We fabricated PLGA/CaSO_4_ scaffolds of different proportions by 3D printing and then evaluated their properties. Physical performance tests showed that adding CaSO_4_ into the PLGA scaffold improved the mechanical properties of the scaffold and made the surface of the scaffold rougher. *In vitro* cytotoxicity experiments showed that PLGA, PLGA/10%CaSO_4_, PLGA/20%CaSO_4_, and PLGA/30%CaSO_4_ groups had good biocompatibility. In addition, the PLGA/20%CaSO_4_ scaffold also promoted the migration of MC3T3-E1 cells. *In vitro* osteogenic experiments showed that PLGA/10%CaSO_4_, PLGA/20%CaSO_4_, and PLGA/30%CaSO_4_ scaffolds had osteogenic properties. Among them, PLGA/20%CaSO_4_ scaffolds significantly promoted the new bone formation *in vitro*. The whisker formed by the combination of CaSO_4_ and H_2_O can make its structure even tighter. The mechanical and degradation properties of PLGA were improved when combined with CaSO_4_ thanks to its tight structure. The rough surface of CaSO_4_ was conducive to cell migration and extension, so PLGA/CaSO_4_ scaffolds could promote cell migration compared with PLGA scaffolds. And CaSO_4_ could also make up for the lack of osteogenic induction activity of PLGA due to its good osteogenesis properties. Therefore, CaSO_4_ could significantly improve the performance of the PLGA scaffold. Among them, PLGA/20%CaSO_4_ showed the best overall performance. In summary, PLGA/20%CaSO_4_ scaffolds were promising for bone tissue engineering applications.

## Data Availability

The original contributions presented in the study are included in the article/Supplementary Material, further inquiries can be directed to the corresponding authors.
