# Supplementary material for: Customized Design 3D Printed PLGA/Calcium Sulfate Scaffold Enhances Mechanical and Biological Properties for Bone Regeneration
Source: Front Bioeng Biotechnol. 2022 Jun 23;10:874931. doi: 10.3389/fbioe.2022.874931 (PMC9260230; doi:10.3389/fbioe.2022.874931)
Supplement: Supplementary file 1 [file DataSheet1.docx]

Supplementary Material

**Customized Design 3D Printed PLGA/Calcium Sulfate Scaffold Enhances Mechanical and Biological Properties for Bone Regeneration**

Tao Liu*^1#^*, Zhan Li*^2#^*, Zehua Chen*^3#^*, Zefeng Lin*^4^*, Binglin Li*^4,5^*, Zhibin Feng*^1^*, Panshi Jin*^1^*, Jinwei Zhang*^1^*, Zugui Wu*^3^*, Huai Wu*^6^*, Xuemeng Xu*^6^**, Xiangling Ye*^3^**, Ying Zhang*^1,6^**

*1 General Hospital of Southern Theater Command of PLA, The First School of Clinical Medicine, Southern Medical University, Guangzhou, Guangdong, PR China, 510515*

*2 Guangzhou University of Chinese Medicine, Guangzhou, Guangdong, PR China, 510006*

*3 The Fifth Clinical College of Guangzhou University of Chinese Medicine, Guangzhou, Guangdong, PR China, 510405*

*4 Guangdong Key Lab of Orthopedic Technology and Implant Materials,* *General Hospital of Southern Theater Command of PLA, Guangzhou, Guangdong, PR China, 510010*

*5 Department of Trauma Orthopedics, Hospital of Orthopedics, General Hospital of Southern Theater Command of PLA, Guangzhou, Guangdong, PR China,510010*

*6 Department of orthopedics, Guangdong Second Traditional Chinese Medicine Hospital, Guangzhou, Guangdong, PR China, 510095*

****Correspondence and requests for materials should be addressed to:***

*Ying Zhang, MD. (email: ying_zhang121@163.com) Department of Trauma Orthopedics, Hospital of Orthopedics, General Hospital of Southern Theater Command of PLA, Guangzhou, Guangdong, PR China, 510010*

*Xiangling Ye, MD. (email:* [*yxl20160228@163.com*](mailto:yxl20160228@163.com)*) The Fifth Clinical Medical College, Guangzhou University of Chinese Medicine, Guangzhou, Guangdong, PR China, 510405*

*XueMeng Xu, MD. (email:* [*xuxuemeng@163.com*](mailto:xuxuemeng@163.com)*) Department of orthopedics, Guangdong Second Traditional Chinese Medicine Hospital, Guangzhou, Guangdong, China, 510095*

***^#^ These authors contributed equally.***


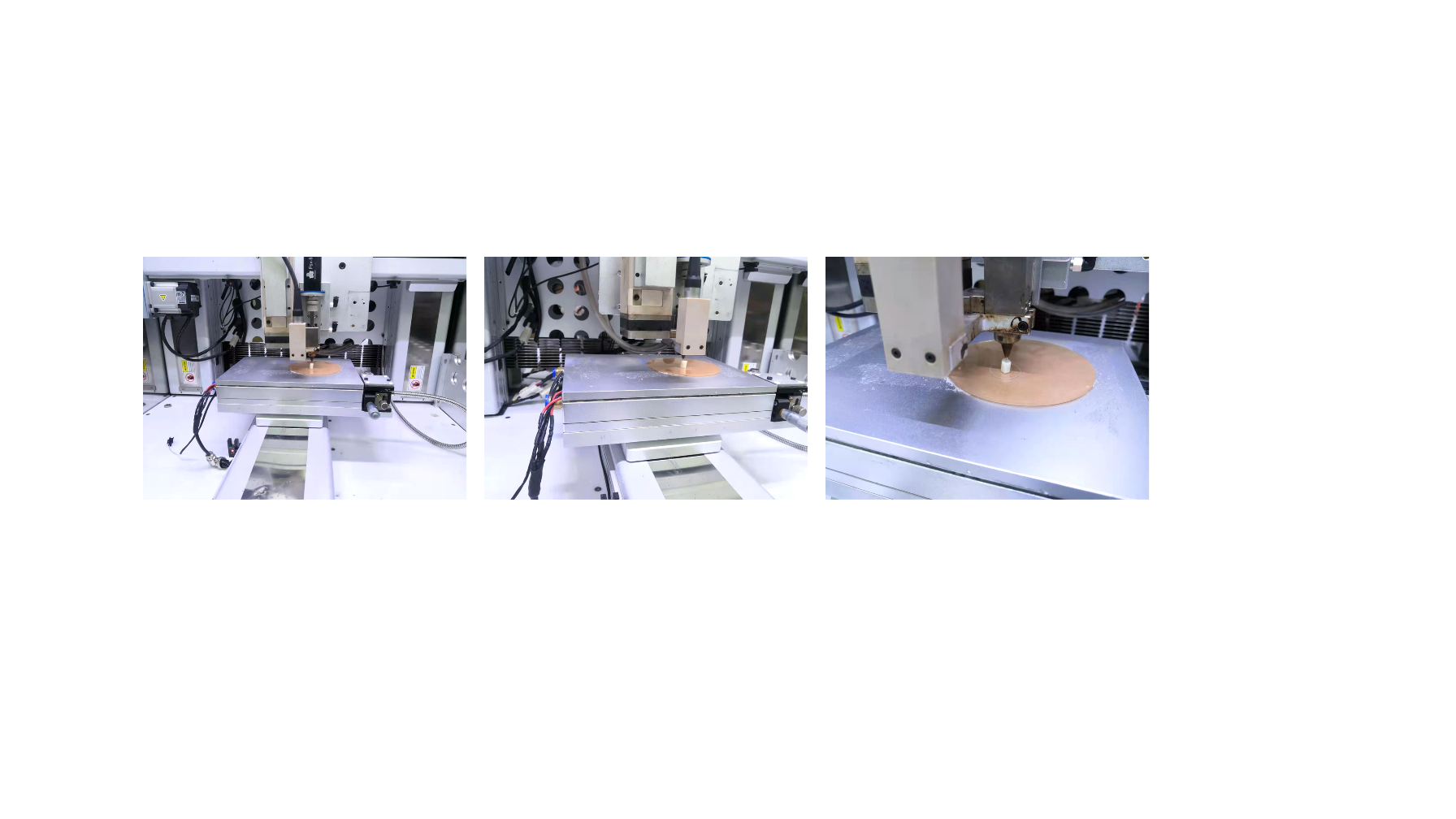


**Figure S1.** Schematic illustration of the fabrication process for PLGA/CaSO_4_ scaffolds by 3D printer.

**
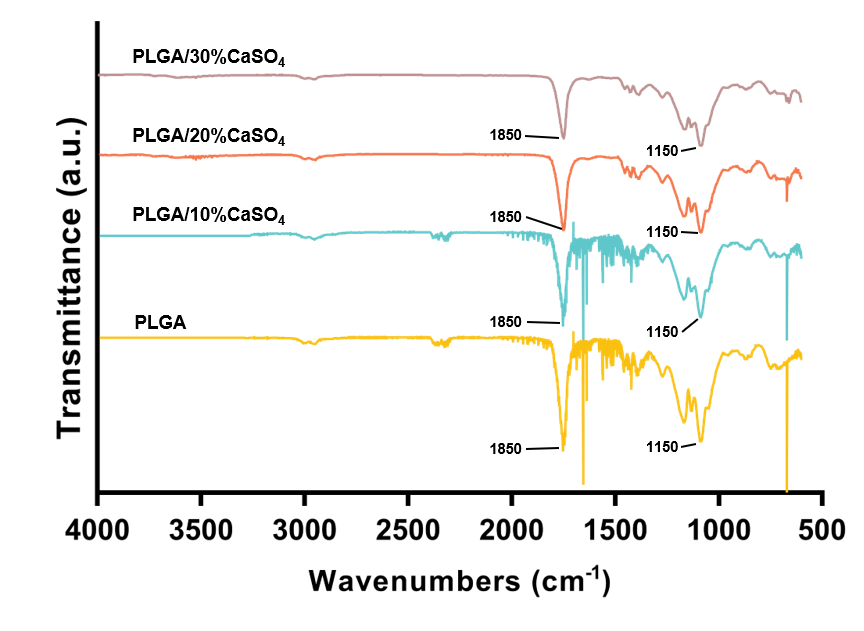
**

**Figure S2.** FTIR spectra of the different scaffolds

**
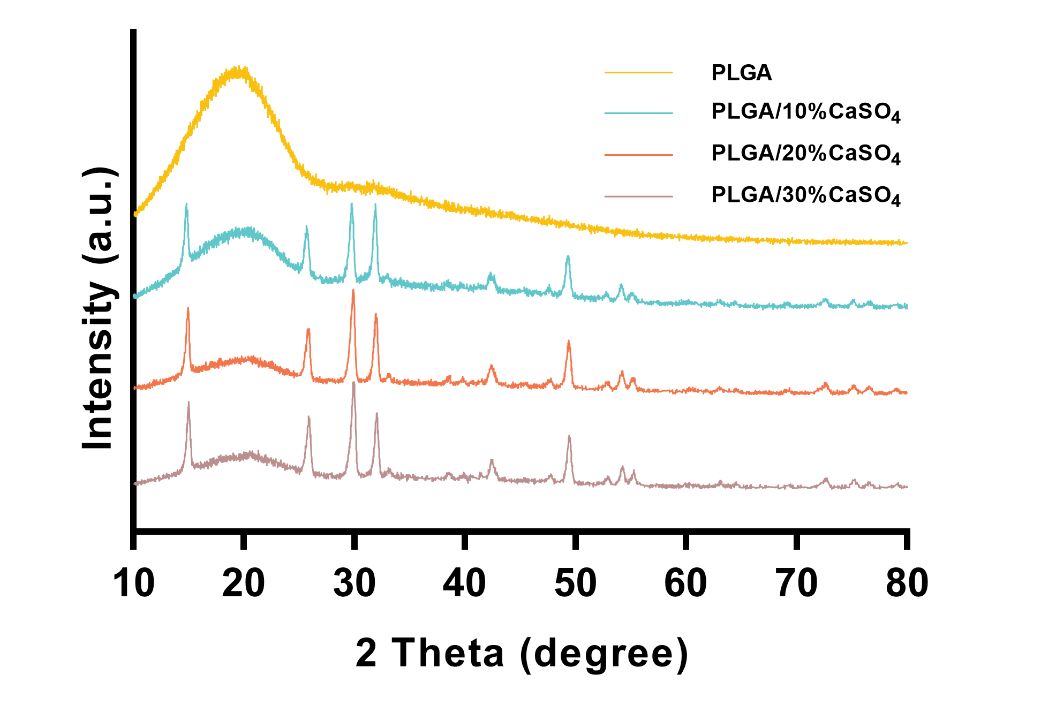
**

**Figure S3.** XRD of the different scaffolds


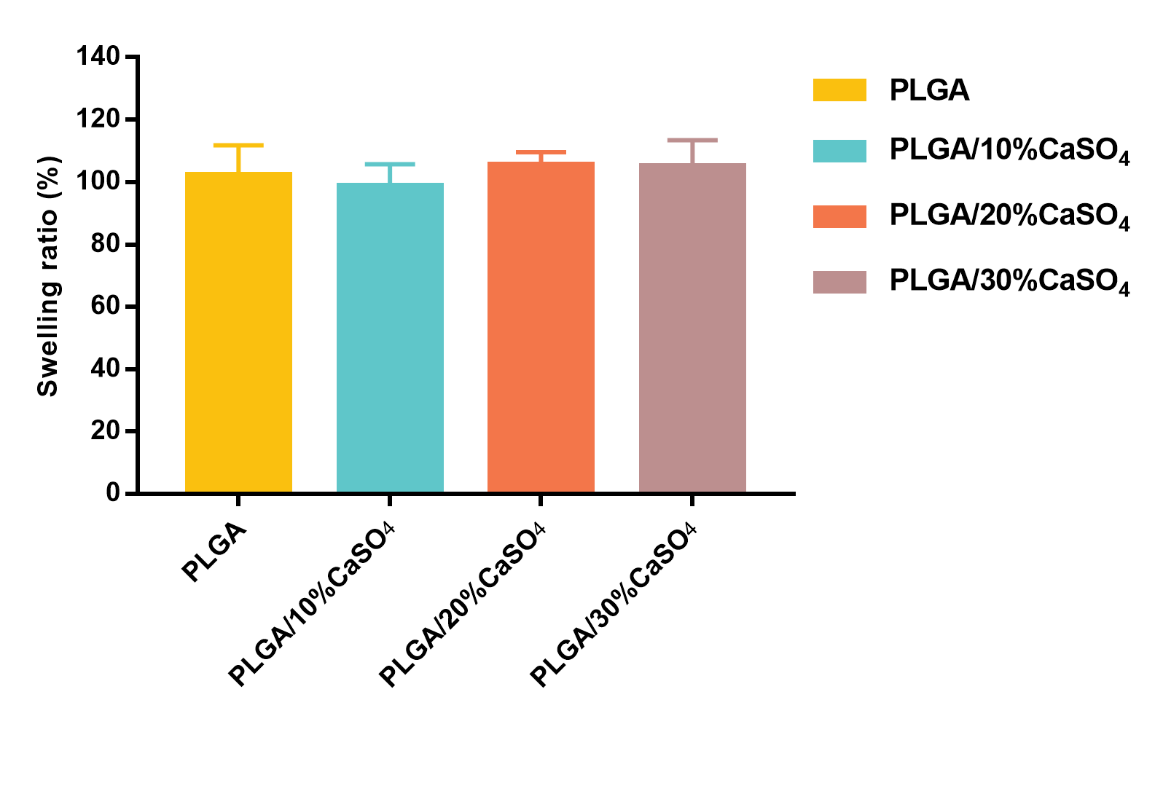


**Figure S4.** The swelling ratio of PLGA, PLGA/10%CaSO_4_, PLGA/20%CaSO_4_, and PLGA/30%CaSO_4_ scaffolds, the swelling ratio of different scaffolds had no statistical difference. Data are presented as mean ± SD (n = 3); **p* < 0.05, ***p* < 0.01, ****p* < 0.001.

**Table S1.** Primers used in RT-qPCR

| Gene | Primer sequences (5′-3′) | | |
| --- | --- | --- | --- |
| β-actin | Forward: | CGAGATTGGCATGGCTTTATTC | |
|  | Reverse: | ACCTTCACCGTTCCAGTTT | |
| RUNX-2 | Forward: | TGGCTTGGGTTTCAGGTTAG | |
|  | Reverse: | GGTTTCTTAGGGTCTTGGAGTG | |
| Col-1 | Forward: | AGACCTGTGTGTTCCCTACT | |
|  | Reverse: | GAATCCATCGGTCATGCTCTC | |
| OCN | Forward: | GGAGCTGCTTTGGTGAGATTAG | |
|  | Reverse: | GAGTAGCCCAGACTACGGATATT | |
| OPN | Forward: | AGTGAGGGTTAAGCAGGAATAC |  |
|  | Reverse: | CAGACTAAGCTAAGAGCCCAAA |  |
| BMP-2 | Forward: | GAACACAAGTCAGTGGGAGAG | |
|  | Reverse: | CACCTGGGTTCTCCTCTAAATG | |
